# Personality traits as protective factors of dementia development

**DOI:** 10.1590/1980-5764-DN-2024-0135

**Published:** 2024-10-25

**Authors:** Laura Beatriz Dias Estrada, Wyllians Vendramini Borelli, Helen Bedinoto Durgante

**Affiliations:** 1 Universidade Federal de Pelotas, Faculdade de Medicina, Curso de Psicologia, Pelotas RS, Brazil.; 2 Universidade Federal do Rio Grande do Sul, Hospital Moinhos de Vento, Department of Morphological Sciences, Porto Alegre RS, Brazil.

**Keywords:** Risk, Dementia, Neuroprotection, Personality, Risco, Demência, Neuroproteção, Personalidade

## Abstract

To date, little is known about psychological and personality variables related to protective factors against the development of dementia. The Five-Factor Model of personality is worldwide recognized and consolidated for understanding the structure and operation of personality, organized into five main factors that delineate and elucidate personality traits/characteristics.

**Objective:**

We aimed to analyze the association of the Five-Factor Model with the epidemiological classification of dementia.

**Methods:**

Cross-sectional design with data collected from the first wave of the Brazilian Longitudinal Study of Ageing (ELSI-Brazil) cohort study, the largest longitudinal study on aging in Brazil. Data gathered from the ELSI-Brazil provided the foundation for demographic and health-related variables (gender, age, education, lifestyle, etc.), mental health indicators, and items associated with personality traits. Logistic regression models were conducted with personality traits as predictors of dementia.

**Results:**

The psychoindicator optimism was the only protective factor associated with dementia (p=0.006). The other variables were not significant predictors in this sample.

**Conclusion:**

Optimism, as a dispositional variable related to personality factors (conscientiousness and neuroticism), emerges as a target variable possible to be developed in preventive longitudinal psychosocial interventions, based on theoretical and empirical evidence of learned optimism, to improve health promotion and self-care throughout life. The data from this study contribute to the advancement of research and to efforts of strengthening services and professional qualification for health and care practices, focused on protective factors, crucial to the global agenda for dementia care and research.

## RESUMO

Até o momento, pouco se sabe sobre os fatores de proteção contra o desenvolvimento de demência relacionados a variáveis psicológicas e de personalidade. O Big Five Factor Model é mundialmente consolidado como uma das principais teorias para compreender a estrutura e o funcionamento da personalidade, composto de cinco fatores principais que elucidam traços/características da personalidade.

**Objetivo:**

Analisar a associação do Big Five com a classificação epidemiológica da demência.

**Métodos:**

Desenho transversal com dados coletados da primeira onda do estudo de coorte Estudo Longitudinal da Saúde dos Idosos Brasileiros (ELSI-Brasil), o maior estudo longitudinal sobre envelhecimento no Brasil. Os dados coletados no ELSI-Brasil foram utilizados para caracterização demográfica, de saúde (sexo, idade, escolaridade, estilo de vida etc.), indicadores de saúde mental e itens relacionados a traços de personalidade. Foram realizados modelos de regressão logística com traços de personalidade como preditores de demência.

**Resultados:**

O traço otimismo foi o único fator protetor associado à demência (p=0,006). As demais variáveis não foram preditoras significativas nesta amostra.

**Conclusão:**

O otimismo como variável disposicional relacionada a fatores de personalidade (conscienciosidade e neuroticismo) pode tornar-se variável alvo a ser desenvolvida em intervenções psicossociais longitudinais preventivas, baseadas em perspectivas teóricas e empíricas de otimismo aprendido para melhorar a promoção da saúde e o autocuidado ao longo da vida. Os dados deste estudo avançam as pesquisas sobre o fortalecimento dos serviços e a qualificação profissional para as práticas de saúde e de cuidado, com foco em fatores de proteção, essenciais na agenda global de cuidado e pesquisa sobre demência.

## INTRODUCTION

The concept of dementia emerged as the characterization of an irreversible neurodegenerative clinical syndrome related to cognitive decline and/or changes in the behavioral level of performance that are important enough to interfere with activities of daily living (functional capacity)^1^. According to Alzheimer’s Disease International (ADI)^2^, around 55 million people live with some type of dementia; the number tends to double, reaching 139 million people by 2050. The ADI^2^ also highlights that there are around 41 million cases of dementia underdiagnosed worldwide, which can result in a drastic increase in the search for services, overloading unprepared public health systems^2^.

Studies have identified risk factors attributed to the development of dementia, such as low education, hypertension, addictive behavior (smoking and high alcohol consumption, obesity, diabetes, depression, traumatic brain injury, social isolation, polygenic risk score (allele Apoeε4, among others), air pollution, deafness, and low nutritional diet^3^. However, research on protective factors for dementia is scarce. To date, little is known about ways to prevent the development of dementia related to psychological variables — referred to here as psychoindicators —, since research has prioritized the biomedical and pharmacological aspect for the management of dementia^4^.

The Brazilian Longitudinal Study of Ageing (ELSI-Brasil)^5^ was conducted on a national scale to understand aspects related to population aging, representing adults aged 50 or over from the general community. With the methodology used by ELSI-Brazil, similar to other international longitudinal studies on aging, it will be possible to compare data internationally and check for possible risk and protective health factors. The database includes items regarding demographic factors, cognition, activities of daily living (functionality), quality of life and well-being, aspects related to mental health and emotional issues such as depression, anxiety, sleep dysfunction, among others^6^. It is worth remembering that, in addition to signaling impairment, subjective cognitive decline and mild cognitive impairment are early stages in the continuum of diseases that develop into future dementia syndromes.^1^

Nevertheless, there are few studies in the scientific literature on risk and protective factors in terms of psychological indicators beyond the genetic and biomedical point of view. In other words, variables capable of influencing the autonomy of individuals and that can be controlled by themselves, such as personality traits and repercussions on lifestyle and behavior, predisposing the individual to higher protection or susceptibility to risks^7^.

Personality may be conceptualized as individuals’ patterns of thoughts, feelings, behaviors, motivation, interpersonal functioning, and ways of experiencing oneself and others^8^. It is generally viewed as a complex dynamic structure shaped by various factors including heritability, physical maturation, early life events and training, identification with significant others, cultural values and roles, critical life experiences and relationships. What is consensus among different theories about the development and structuring of personality is that it is capable of, to some extent, determining/predicting an individual’s behavior^9^.

In terms of theory for understanding personality, the Five-Factor Model^10^, also known as the Big Five, is presented as a model for understanding its structure and functioning, composed of traits (pattern of thoughts, emotions and behaviors consistent over time), also called facets, which make up five major personality factors, namely: neuroticism, extraversion, openness to experience, agreeableness, and conscientiousness. In terms of health, knowing about possible interrelationships of personality traits/factors can potentially lead to a wider array of actions related to health promotion and disease prevention. Factors like neuroticism include characteristics that indicate a more intense form of psychological suffering, emotional instability, and vulnerability; high scores have been associated with anxiety and depression and used to screen and identify individuals with a higher propensity to develop psychopathology^11^. Conscientiousness is another factor strongly related to health care behaviors; high conscientiousness scores tend to represent a disciplined person, more careful with their health, and, therefore, tending to live a long life^12^.

In Brazil, the Personality Factor Battery was developed^12^ based on the Big Five model, preserving the five factors proposed in the original model, and containing culturally adapted items. The classification and translation of the factors were: neuroticism, extraversion, socialization (agreeableness), achievement (conscientiousness), and openness (openness to experiences).

In this context, given the lack of national literature regarding possible psychological indicators of risk and protection for dementia, this study aimed at analyzing the association of the Five-Factor Model and the epidemiological classification of dementia. To this end, personality traits were empirically analyzed, in addition to the genetic and biomedical points of view, as possible risk and protective factors for dementia.

## METHODS

### Design

This study used data collected by the ELSI-Brazil. The ELSI-Brazil used an inverse sampling design^13^, which allowed researchers to define the number of units needed to be examined. The method was applied in ELSI-Brazil through sequential visits to previously established households. At the end of the collection, the sample was estimated at 10 thousand participants living in 70 municipalities, located in major geographic regions of the country^6^.

### Participants

Data from 9,412 participants from the ELSI-Brazil database referring to the first part of the program (2015–2016) were used.

### Instrument

The instrument used was the ELSI-Brazil Individual Interview Questionnaire (available at http://elsi.cpqrr.fiocruz.br/questionario/) (attachment). From this, items regarding demographic and health characterization (gender, age, education, lifestyle, etc.) were selected based on scientific literature, as well as items indicating mental health and/or related to personality traits/factors based on items from the Personality Factor Battery^12^.

Cognitive function was assessed using a test battery (immediate memory, semantic fluency, and delayed memory scores), an initiative known as *The Health and Retirement Family* ^14^. This specific design of the ELSI-Brazil cognitive function module allows for direct comparison of Brazilian results with results found in other countries, such as China (The China Health and Retirement Longitudinal Study), the United States (The US Health and Retirement Study), England (The English Longitudinal Study of Ageing), Mexico (Mexican Health and Aging Study), among others.

For depression indicators, the ELSI-Brazil contains eight items from The 8-item Center for Epidemiologic Studies — Depression Scale (CES-D-8)^15,16^. In addition, it includes items to measure skills related to simple or advanced activities of daily living, based on the Lawton Instrumental Activities of Daily Living Scale (IADL) and Katz Index of Independence in Activities of Daily Living (KATZ-ADL for functionality^17^.

To classify subgroups with ‘probable dementia’ or ‘not probable dementia’, scores from the Katz functionality scale were used, with a value considered to be less than 6 points in the sum of the items for functional loss; CES-D-8 scale for depression items; item scores from tests of cognitive function for cognitive decline, specifically the semantic fluency test as a cognitive screening^18^. An individual who presented cognitive decline (below the cut-off point for the semantic fluency test) and also loss of functionality (below the cut-off point for the Katz scale) was defined as having ‘probable dementia’. All values were adjusted for age and education.

### Procedures and ethical considerations

According to information collected directly from the ELSI-Brazil platform, the study follows all the resolutions of the National Health Council required for scientific studies with human beings, such as 196/1996 and its complements, including 292/1999, 340/2004, 346/2005, 347/2005 and 466/2012. The ELSI-Brazil was approved by the Ethics Committee of the Oswaldo Cruz Foundation — Minas Gerais and its process is registered on Plataforma Brasil (Certificate of Presentation for Ethical Appreciation — CAAE: 34649814.3.0000.5091).

The sample was collected to represent the non-institutionalized Brazilians aged 50 or over. For stratification and better selection of areas for research, the geographic operational base of the Brazilian Institute of Geography and Statistics^19^ was used, thus ensuring that the sample includes urban and rural areas of small, medium, and large municipalities. These were separated into four strata according to population size. To decide the size of the municipality and the number of municipalities assigned to each stratum, the stratum construction method of Lavallée and Hidiroglou^20^ was carried out using the module for stratification of the R package (R Foundation for Statistical Computing, Viena, Austria). Therefore, the four strata were categorized into: first stratum (≤26,700 inhabitants from 4,420 municipalities), second stratum (26,701–135,000 inhabitants from 951 municipalities), third stratum (135,001–50,000 inhabitants from 171 municipalities), and fourth stratum (>750,000 inhabitants from 23 municipalities).

All participants signed informed consent forms for each of the research procedures. Only one adult from each household was invited to respond to the household interview, and all residents aged 50 years or over were invited to participate in the individual interview and the physical measurements. In this study, only psychoindicators are evaluated without using biomarkers. The interviewers were trained and certified before the start of fieldwork. To guarantee the confidentiality of data obtained in interviews and other procedures, the information was archived without nominal identification and is used only for scientific research purposes.

### Statistical analysis

This analysis used ELSI-Brazil variables related to personality traits, as mentioned previously. Initially, items were selected and grouped into two models for logistic regression analysis, one containing items based on risk factors for the probable development of dementia (S15+S17+S37+S39+S40+N3+N73+P73+P78+P79+P83), and another with items representing protective factors (S38+S42+S45+S46+S47+S48+S49+S51+S52+S53+S54). The R software (R Foundation for Statistical Computing, Viena, Austria, V4.1.0) was used. After initial analysis of the database and to standardize the results, items were dichotomized due to the high variation in results in measurements at a scalar level. Thus, for example, questions included in each model with varying response options were dichotomized as follows: (1) never, (2) sometimes, (3) always; the alternatives (2) sometimes and (3) always were combined.

Participants were separated into “probable dementia” and “probable not dementia” groups, with the differentiation between the two made through the assessment of cognitive criteria and functionality criteria. The cognitive decline criterion was based on the semantic fluency test with animals. The cut-off point was adjusted for age and education, with the individual classified as declining when scored below the cut-off point^16^. Functionality was measured using the Katz scale, in which a score below 6 classifies the individual as having lost functionality^21^. The Katz scale was defined as the sum of the following variables: P40+P43+P46+P17+P59+P58 from the ELSI questionnaire.

In a second analysis of the database, overlapping results were found between participants with ‘probable dementia’ and ‘probable depression’, considering that depression in early life is a risk factor for dementia, and depression in old age can be seen as a precursor of dementia^22^. After the theoretical review, participants with high depression scores were removed to avoid confusing depressive symptoms with the probability of dementia and to ensure greater robustness of the models. The exclusion criterion was based on the sum of the CES-D-8 items (R2+R3+R4+R5+R6+R7+R8+R9), with a cut-off point equal to 4^23^. Data are presented as mean (±standard deviation — SD), and a p-value <0.05 is considered statistically significant.

## RESULTS

Individuals with severe depressive symptoms were excluded from the analysis. The total number of participants who entered the models for statistical analysis was 5,045, of which 59.7% women and 40.3% men, with a mean age of 69.68 (SD=7.45). The average level of education in years prevailed at 4.63 (SD=4.21). Regarding distribution by regions: 10% Central-West, 7.3% North, 26.9% Northeast, 13.5% South, and 42.2% Southeast. As for races, the distribution of participants was 1.1% yellow, 39.6% white, 2% indigenous, 42.9% brown, and 9.8% black Table 1. When analyzing all items in the logistic regression models, the only predictor of dementia was (S54) ‘the frequency of feeling optimistic about the future’ (OR 0.53, 95%CI 0.33–0.84, p=0.006), adjusted for age and education Table 2, Table 3 and Table 4.

**Table 1:** Final sample characteristics.

	Dementia (n=122)	Cognitively unimpaired (n=4,752)	p-value
Age (years)	73.1 (±8.2)	69.5 (±7.4)	<0.0001
Sex (M)	51 (41.8%)	1,929 (40.6%)	0.080
Education, mean years (SD)	5.0 (±4.0)	4.7 (±4.2)	0.021
Family income, mean minimum wages (SD)	4.5 (±2.8)	4.7 (±3.4)	0.014
CES-D-8 mean scores (SD)	1.6 (±1.1)	2.6 (±2.3)	<0.0001
Region (%)			
Midwest	7 (5.7)	485 (10.2)	0.59
North	10 (8.2)	346 (7.3)	
Northeast	28 (23.0)	1,280 (26.9)	
South	19 (15.6)	641 (13.5)	
Southeast	58 (47.5)	2,000 (42.1)	
Race (%)			
Asian	1 (0.9)	52 (1.1)	0.06
White	55 (48.7)	1,893 (41.7)	
Indigenous	0 (0.0)	98 (2.2)	
Brown	49 (43.4)	2,034 (44.8)	
Black	8 (7.1)	467 (10.3)	

Abbreviations: SD, standard deviation.

**Table 2:** Model applied for protective factors.

Protective factors	Estimate	Std. Error	t-values	p-value
Intercept	−9.26	1.27	−7.28	<0.001
Age	0.07439	0.01	5.18	<0.001
Education	0.03	0.03	1.3	0.2
S38 How often do you feel free to make plans for the future?	−0.32	−0.22	−1.44	0.15
S42 How often are you able to pursue activities that give you pleasure?	0.24	0.24	0.98	0.32
S45 How often do you look forward to each day with enthusiasm?	0.19	0.28	−0.66	0.5
S46 How often do you feel like your life has meaning?	−0.03	0.35	−0.11	0.91
S47 How often do you enjoy the things you do?	−0.05	0.53	−0.1	0.92
S48 How often do you like to be in the company of other people?	0.32	0.38	0.86	0.39
S49 How often do you feel happy when thinking about what you have experienced?	−0.14	0.30	−0.46	0.63
S51 How often do you like to do new things?	−0.11	0.28	−0.42	0.67
S52 How often do you feel satisfied with your achievements?	0.42	0.39	1.07	0.28
S53 How often do you think life is full of opportunities?	0.51	0.35	1.4	0.14
S54 How often do you feel optimistic about the future?	−0.63	0.23	−2.73	0.006

**Table 3:** Model applied for risk factors.

Risk factors	Estimate	Std. Error	t-values	p-value
Intercept	−4.18	3.5	−1.19	0.23
Age	0.005	0.04	0.12	0.9
Education	0.06	0.06	0.9	0.32
S15 Do you feel uncomfortable because you think people try to help you more than you think you need?	0.33	0.69	0.47	0.63
S37 How often do you feel that things that happen to you are beyond your control?	−0.26	0.56	−0.5	0.64
S39 How often do you feel excluded from events?	0.27	0.71	0.37	0.7
S40 How often can you do the things you want?	−1.12	0.61	−1.8	0.07
N3 In the LAST 30 DAYS, for how many days was your mental health not good, i.e., did you feel depressed, stressed, or had emotional problems?	0.01	0.01	0.85	0.39
N73 In the LAST WEEK, how often did carrying out your routine activities require a lot of effort from you?	0.94	0.59	1.6	0.11
P73 In the LAST 12 MONTHS, have you gone out with other people to public places (restaurants, movies, clubs, squares, etc.)?	−0.08	0.55	−0.16	0.87
P78 In the LAST 12 MONTHS, did you get together with your colleagues/friends to play (checkers, chess, cards, dominoes, billiards, etc.)?	−0.9	0.61	−1.49	0.13
P79 In the LAST 12 MONTHS, have you done manual work or practiced any hobbies such as painting, sculpture, drawing, embroidery, knitting, crocheting, gardening, horticulture, etc.?	−0.05	0.45	−0.12	0.9
P83 In the LAST 12 MONTHS, how often did you do volunteer work?	0.08	0.47	0.18	0.86

**Table 4:** Items assessed as risk or protective factors for dementia.

Variables	OR	t-values	CI	p-value
Age	1.08	5.178	1.04–1.10	0.07
Education	1.03	1.276	0.98–1.09	0.203
S38 How often do you feel free to make plans for the future?	0.72	−1.441	0.46–1.12	0.150
S42 How often are you able to pursue activities that give you pleasure?	1.27	0.980	0.78–2.07	0.327
S45 How often do you look forward to each day with enthusiasm?	0.82	−0.667	0.46–1.45	0.505
S46 How often do you feel like your life has meaning?	0.96	−0.110	0.48–1.91	0.912
S47 How often do you enjoy the things you do?	0.94	−0.102	0.33–2.69	0.919
S48 How often do you like to be in the company of other people?	1.38	0.860	0.65–2.90	0.390
S49 How often do you feel happy when thinking about what you have experienced?	0.86	−0.469	0.47–1.58	0.639
S51 How often do you like to do new things?	0.88	−0.425	0.50–1.54	0.670
S52 How often do you feel satisfied with your achievements?	1.52	1.071	0.70–3.32	0.285
S53 How often do you think life is full of opportunities?	1.66	1.453	0.83–3.33	0.147
S54 How often do you feel optimistic about the future?	0.53	−2.731	0.33–0.83	0.006*
S-15 Do you feel uncomfortable because you think people try to help you more than you think you need?	1.39	0.473	0.34–5.54	0.636
S-37 How often do you feel that things that happen to you are beyond your control?	0.76	−0.464	0.25–2.35	0.643
S-39 How often do you feel excluded from events?	1.30	0.378	0.32–5.34	0.706
S-40 How often can you do the things you want?	0.32	−1.818	0.09–1.10	0.071
N-3 In the LAST 30 DAYS, for how many days was your mental health not good, i.e., did you feel depressed, stressed, or had emotional problems?	1.01	0.858	0.98–1.04	0.392
N-73 In the LAST WEEK, how often did carrying out your routine activities require a lot of effort from you?	2.57	1.608	0.80–8.24	0.110
P-73 In the LAST 12 MONTHS, have you gone out with other people to public places (restaurants, movies, clubs, squares, etc.)?	0.91	−0.159	0.30–2.75	0.873
P-78 In the LAST 12 MONTHS, did you get together with your colleagues/friends to play (checkers, chess, cards, dominoes, billiards, etc.)?	0.40	−1.489	0.12–1.34	0.138
P-79 In the LAST 12 MONTHS, have you done manual work or practiced any hobbies such as painting, sculpture, drawing, embroidery, knitting, crocheting, gardening, horticulture, etc.?	0.94	−0.119	0.42–2.80	0.905
P-83 In the LAST 12 MONTHS, how often did you do volunteer work?	1.08	0.180	0.42–2.80	0.857

Note: *p<0.01.

Abbreviations: OR, odds ratio; CI, confidence interval.

## DISCUSSION

This study aimed at analyzing the association of the Five-Factor Model with the epidemiological classification of dementia. The analysis was carried out based on general items from the questionnaire of the national epidemiological survey ELSI-Brazil (2015–2016), which indicated correspondence to the items in the Factorial Personality Battery. The item regarding optimism was the only protective factor with statistical significance (p=0.006), as a possible predictor of dementia. The other variables were not significant predictors in this sample.

Optimism may be defined, according to the theory of learned optimism, the attribution of causality, the interpretation of positive or negative life events^24^. Individuals with higher optimism scores tend to attribute the justification or cause of a failure, taking into account external factors and not only internal ones^25^. Also, according to Rashid and Seligman^25^, these individuals can assign causes to negative events as isolated instead of having repercussions on all areas of their lives and perceive failure as temporary, not permanent. Optimism is, in essence, a cognitive process, i.e., a path towards desired objectives, a systematic change of analysis about the events crossing the path, and which results or not in setting achievable goals working towards its achievement, believing that a good future is something that can be promoted^25^.

Considering the Big Five theoretical model, optimism is more specifically related to two personality factors: conscientiousness and neuroticism. Throughout life, conscientiousness is present in activities involving prudence and self-care^25^. Prudence is about being careful when making choices with discernment and prioritization, both in terms of saying and doing something that the person may regret in the future. It is related to both zeal and excess or carelessness to have a fuller life^26^. Self-care refers to taking care of themselves in all areas of life, whether physical, mental/emotional, social, or spiritual. Relating in a practical way to the possible development of dementia, prudence and self-care are indispensable tools in making decisions regarding health, especially risk behaviors, which are related to dementia syndromes on a longitudinal basis, such as eating styles, undergoing medical check-ups, substance abuse (high alcohol consumption, smoking, among others), and sedentary lifestyle. In this sense, investing efforts in health promotion practices that include aspects of self-care and prudence throughout the life cycle can be essential for preventive science in order to mitigate costs to public health systems for the future management of dementia.

Neuroticism, at high levels, tends to result in more intense psychological suffering, likely causing depressive and anxiogenic symptoms^27^. Studies suggest that individuals with high neuroticism scores have greater reactivity to stress, which, over time, can make them more prone to cognitive decline or the development of neurodegenerative diseases and/or psychopathologies^28,29^. It is worth remembering that clinical depression, major depression, when not treated throughout life, is a risk factor for dementia with the possibility of early prevention^3^. Such characteristics related to high neuroticism are opposite poles from those observed in people with higher results in optimism, and, as a result, the latter tend to demonstrate and reflect lower levels of stress and depression^25^. Therefore, investing in strategies, interventions, and programs that focus on the development of optimistic cognitive processes may prove to be prophylactic for long-term cognitive impairments^30^, reducing negative repercussions on health and life^31^.

Similarly, it is also possible to empirically investigate the relationship between the development of dementia syndromes and other personality factors, such as openness to experience, which refers to exploratory behavior and recognizing the importance of having new experiences, and the construction of new values along life^25^. Regarding health as a possible ‘value’ acquired and altered throughout life, openness may become an important mediator in the process of adjusting new behaviors for better health. Individuals with high levels of openness, however, may exhibit low motivation for repetitive activities and may easily become bored^25^. On the other hand, when in line with higher levels of conscientiousness (motivation to achieve goals, perseverance, consistency, planning, organization, punctuality), higher levels of openness may help individuals become more inclined to adhere to new habits and different health-related activities and practices^25^. This may be particularly important to perform daily tasks that require long-term repetition, such as physical activity, good nutritional diet, and overall health care.

Additionally, considering the relationship between personality traits and cognitive resilience, a mechanism capable of making cognitive adaptations to the natural or pathological changes of aging, maintaining good cognitive and functional performance through compensation^32,33^, it reinforces the need for investments in health promotion interventions that address these aspects. The development of psychological strengths/potentialities results in greater flexibility to deal with expected declines, exercising/formulating cognitive resilience^34^. Furthermore, according to Hill and Smith^35^, the aging of those who develop it in a positive and optimistic way can be distinguished through the following aspects: social skills and psychological strengths/potentialities; cognitive and behavioral flexibility; decision-making capacity with a view to psychological well-being, especially toward irreversible losses; and an optimistic perspective about themselves and their moment in life.

Finally, considering the lack of a current national plan for dementia management, it is essential to highlight and act on factors of protection. Also, regarding the lack of continuous support and palliative care to informal/family caregivers, the overwhelming majority in terms of those who provide care for people with dementia in Brazil (pre- and post-diagnosis, mourning, end of life, quality of life of these individuals), strengthening services, qualifying health professionals with protocols to guide practices centered on the promotion of protection factors are indispensable in the national health agenda^36,37^. Evidence indicates that these measures can reduce the incidence of mental disorders and the emotional impact on family members, caregivers, and people involved in dementia management, therefore reducing health costs^31,38,39^.

Aiming for the integration of health and care services and long-term sustainability and autonomy in health management, the implementation of programs focused on health promotion emerges as a robust option for health systems. In Brazil, an example of such practice may be the ‘Vem Ser’ program developed in 2016, which is grounded in theoretical perspectives of Cognitive-Behavioral Therapy^40^ and Positive Psychology^35,41^. The program aims, through the development or strengthening of protective factors (strengths/virtues), to foster one’s autonomy and accountability towards a healthier lifestyle and health processes^37^. The program consists of six structured weekly sessions, two hours each, conducted in groups, and adapted into two versions: in-person (for retirees) and online (for health, education, and care professionals, retired or active in the labor force)^42^. Considering the low costs of the program, its effective outcomes and benefits for health gains, and current manualized versions available for training of group facilitators, it became an option for health promotion in the current economic and social landscape of Brazil^43,44^.

Another point worth noting in that the multifaceted and complex nature of personality can pose challenges in controlling the generalization of results when designing and implementing psychosocial interventions for health promotion; a process that requires scientific rigor in the development and application of treatment protocols. A culturally sensitive intervention is deemed imperative to produce desired outcomes and reduce the risks of iatrogenic effects. Health is influenced by multifactorial aspects, including personality traits as potential health determinants. All these factors tend to impact public health when the broader context is not considered. Thus, public health investments are compensated when applied under adequate implementation conditions and with evidence based on reducing mental health problems^45^. Additionally, it is worth considering work on personality traits as target variables in early to moderate stages of dementia as well as in preventive science and longitudinal health promotion practices.

This study presents a limitation arising from the inclusion of items derived from questionnaires other than the original Personality Factor Battery. Despite the correspondence and semantic similarity of the items, in an ideal scenario, the sample would have responded to the Personality Factor Battery, thereby achieving greater precision in the analysis of the data and variables included in this study.

In conclusion, the optimism indicator item remained the only protective factor for dementia in this sample. This indicator was related to conscientiousness and neuroticism, and, with knowledge of its impacts on overall health, optimism as a guide towards the goals of each individual and filter that makes the future more positive by giving the impulse necessary to work in its realization, it is necessary to think of strategies to promote and exercise such aspects. Therefore, as optimism is related to factors and personality traits and is changeable, it is possible to learn optimistic perspectives and train the brain to analyze facts and events from a more favorable perspective for health promotion and self-care throughout life and the promotion of healthy aging. Thus, interventions for health promotion based on optimism development can be pertinent and prophylactic for the prevention of dementia syndromes and also offer support for caregivers and professionals through structured protocols on the management and confrontation of adversities due to dementia.
